# Combined dental wear and cementum analyses in ungulates reveal the seasonality of Neanderthal occupations in Covalejos Cave (Northern Iberia)

**DOI:** 10.1038/s41598-019-50719-7

**Published:** 2019-10-04

**Authors:** Carlos Sánchez-Hernández, Lionel Gourichon, Eric Pubert, William Rendu, Ramón Montes, Florent Rivals

**Affiliations:** 1 Institut Català de Paleoecologia Humana i Evolució Social (IPHES), Zona Educacional 4, Campus Sescelades URV (Edifici W3), 43007 Tarragona, Spain; 2 Universitat Rovira i Virgili (URV), Àrea de Prehistòria, Avinguda de Catalunya 35, 43002 Tarragona, Spain; 3 Université Côte d’Azur, CNRS, CEPAM, Nice, France; 4 PACEA (CNRS-UMR 5199), Université de Bordeaux, Bâtiment B2, Allée Geoffroy Saint Hilaire, Pessac, France; 5 Itinerario Cultural del Consejo de Europa Prehistoric Rock Art, Madrid, Spain; 6 ICREA, Pg. Lluís Companys 23, 08010 Barcelona, Spain

**Keywords:** Archaeology, Palaeoecology

## Abstract

We propose for the first time the use of the combination of two high-resolution techniques, dental wear (meso- and microwear) and dental cementum analyses, to gain a better understanding of Neanderthal subsistence strategies and occupational patterns. Dental wear analysis provides information not only on ungulate palaeodiet and palaeoenvironments but also on hunting time and seasons. Dental cementum analysis allows the accurate determination of the age and season at death of a prey. Our study has focused on the Cantabrian region and has applied both methods to investigate the Mousterian faunal assemblages in Covalejos Cave. Identification of the ungulate palaeodiet reveals information on the environmental conditions of the studied region. Moreover, it may facilitate observation on the evolution of both palaeodiet and palaeoenvironment throughout the site sequence. Results show a general stability in the palaeoenvironmental conditions and in the ungulate palaeodiet throughout the Mousterian sequence; this finding may be attributed to the role of the area as a climate refuge, and slight differences in levels 8, 7 and 4 suggest long- or short-term but repeated Neanderthal occupations at different seasons in the annual cycle.

## Introduction

The hunter–gatherer groups that inhabited Europe during the Middle Palaeolithic (MIS 5–3) were subject to strong, rapid climatic changes^1,2^ that altered their environment and consequently their subsistence resources. One of the most important issues that human groups needed to deal with is their organization and settlement selection. Among the factors and requirements that influence their choice and duration of settlements include the various subsistence strategies of these human groups, the stability of the environment, and the prevailing local conditions. That is, the availability of abiotic and biotic resources, such as raw material accessibility, prey distribution, feeding strategies, and seasonal movements^3–6^. Neanderthal groups living in Europe at that time were characterized by high mobility throughout their territory and by a wide variety of settlement types, ranging from butchering sites or raw material-rich sites (short-term/seasonal occupations)^7–11^ to base/residential camps (long-term occupations)^11–15^. Therefore, the characteristics of various environments and the needs arising from group subsistence influenced the strategic planning of Neanderthal groups as regards their choice of settlement type and duration of settlement^4,6,16–20^.

Traditionally, mobility and settlement patterns were assessed using classical approaches, such as zooarchaeological or lithic studies^21–27^, which are occasionally complemented by methods employed at different temporal scales^17,25,28–32^. All these methods provide high-resolution data based on the study of specific animal skeletal parts. These data are linked to biological processes, such as dental eruption patterns^33,34^, degree of crown height attrition^35–37^, and seasonal growth bands of dental cementum^38–42^; to individual features linked to the diet as revealed by analysis of stable carbon and oxygen isotopes^30,43,44^ or dental wear^45,46^. However, accurate data are usually difficult to obtain from the archaeological records with the use of a single method^47^; when used independently, a certain technique may provide data of low resolution given that several variables may have been involved during the formation of archaeological evidences. Thus, the use of dental wear techniques alone do not provide a comprehensive picture that allows one to appreciate minimal variations in the seasonality of human settlement patterns.

Applying these methods to archaeological contexts for the first time, we proposed herein to combine two high-resolution techniques to obtain accurate seasonal estimates of human occupation; these techniques are the dental wear (meso- and microwear) analysis^37,45^ and the dental cementum analysis^38,39,48–50^. We applied both methods on a series of ungulate molars (obtained from *Cervus elaphus, Equus ferus, Bos/Bison*).

Dental wear meso- and microwear techniques respond to different types of temporal resolutions for a certain parameter (see Methods). When these two dental wear techniques are combined, the information will reflect not only the degree of attrition and abrasiveness of available resources in an annual cycle (mesowear), but also any possible variations due to seasonal environmental influences (microwear)^51^. Mesowear analyses investigate such macroscopic features as the shape of cusps and provides a long-term signal that covers months to years^37^. By contrast, microwear analyses investigate the microscopic features on an occlusal surface caused by food items and provides a short-term signal that covers days to weeks before death^52^. These two dietary signals in ungulates allow the identification of seasonal pattern of prey acquisition (i.e. in ungulate mortality events)^51^. These signals might reflect both the general and immediate ungulate behaviour and the general and seasonal environmental conditions around sites, as well as reflect the seasonality of human occupations in those locations^7,11,17,28,29,32,51^. These methods use two different temporal scales that should be understood within the context of an individual’s life time. In fact, a macroscopic wear observed through mesowear analysis requires several months (longer than a natural season, i.e. >6 months) to become evident, and this could be considered a long temporal scale within an animal’s life time^37,53^. By contrast, microscopic features produced by feeding particles interacting with dental enamel appears and are replaced within days/weeks; thus, they are considered short temporal scale within an animal’s life time^52^. Note that the concepts of ‘season’ and ‘seasonality’ within the framework of the information derived from dental wear refer to the duration of mortality events and not to a natural season thereof (e.g. spring, summer…).

The second method, which is based on the analysis of microscopic growth marks within the dental cementum (see Methods), provides two kinds of information about the animal being studied. The number of cementum increments reveals the age at death, whereas the nature of the last increment and its degree of development indicate the season when the animal was killed^39,48,49,54–58^. Such data reveal the seasonality of hunting episodes of the Neanderthal groups and therefore offer some important clues on the period and duration of settlement in an annual cycle. The acquisition of such information lies in the growth pattern of the type of cement on which this research focuses, namely acellular extrinsic fibre cementum (also called acellular cementum, AC)^59,60^. In fact, an AC shows a regular growth pattern involving an alternating rapid and slow collagen deposition and mineralisation phases throughout an annual cycle. This varying rate of collagen deposition and mineralization throughout the annual cycle produces two types of increments that correspond to different times of the year (see Methods)^39,40,48,55–64^. The technique employed is partially destructive because one of the roots of a tooth is used to produce thin sections. Several thin sections are obtained from each root to obtain a correct count of cementum increments. These thin sections are made thinner until they are translucent so that the growth marks can be observed through a polarizing light microscope^40,41,48,60,65,66^. This technique reveals the natural season when the mortality and feeding events took place. This technique is especially useful when variations in feeding pattern have a short duration, allowing the correlation of the attritive/abrasive properties of a consumed resource with the natural season in an annual cycle (e.g. spring, summer…).

**Figure 1:**
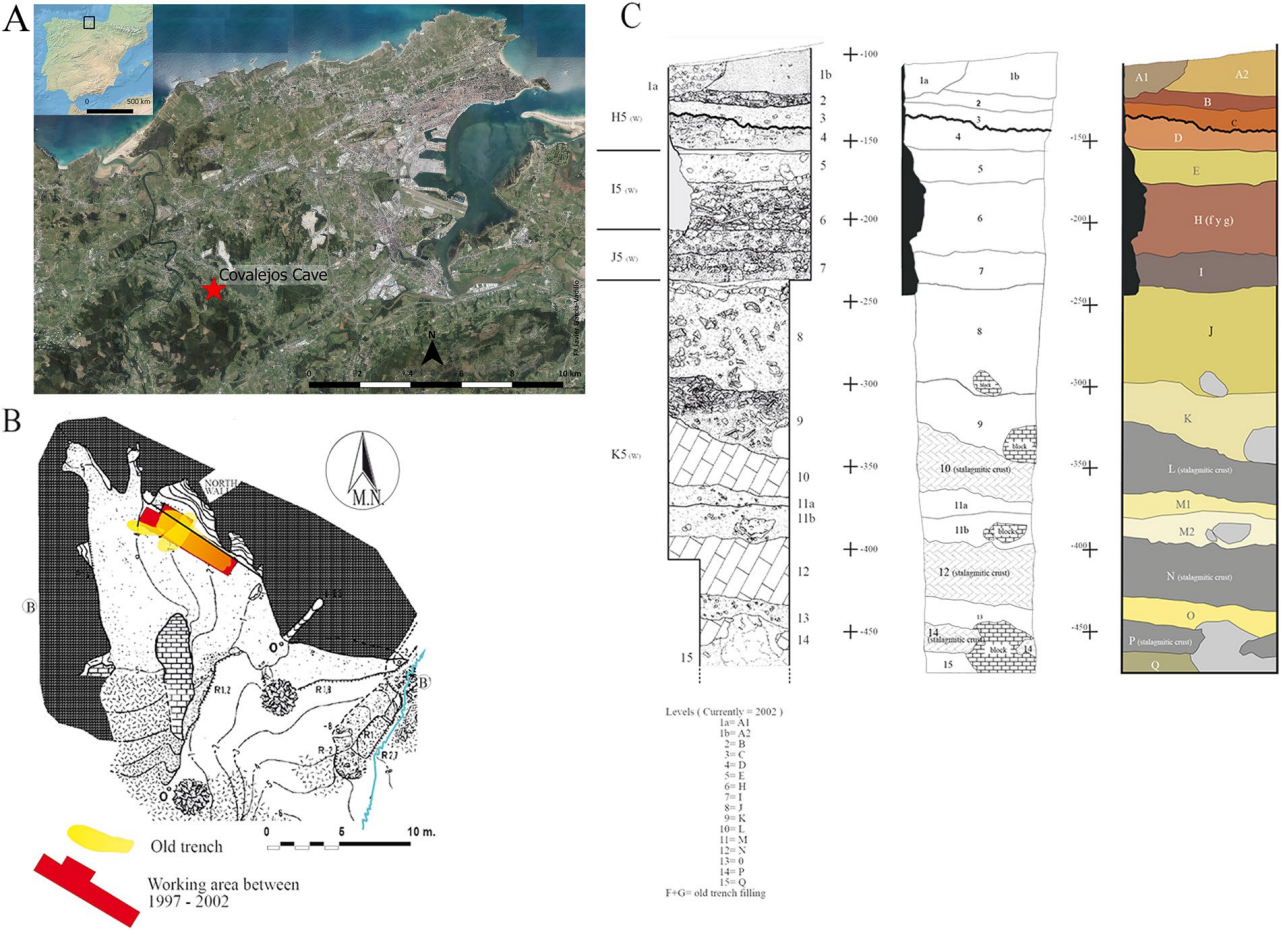
Location (**A**), topography (**B**) and stratigraphy (**C**) of Covalejos Cave^78^. Map of the Iberian Peninsula prepared using Natural Earth (Free vector and raster map data @ naturalearthdata.com) and modified in InkScape by Francisco Javier García Vadillo. Aerial map of the Santander area prepared using the free software QuantumGis 2.14.2 [QGIS Development Team (2014). QGIS Geographic Information System. “Open Source Geospatial Foundation Project”. http://qgis.osgeo.org] and obtained from PNOA (Plan Nacional de Ortofotografía Aérea, pp. 34–35), which is available at the open source Spanish CNIG (Centro Nacional de Información Geográfica: http://centrodedescargas.cnig.es/CentroDescargas/index.jsp#).

**Figure 2:**
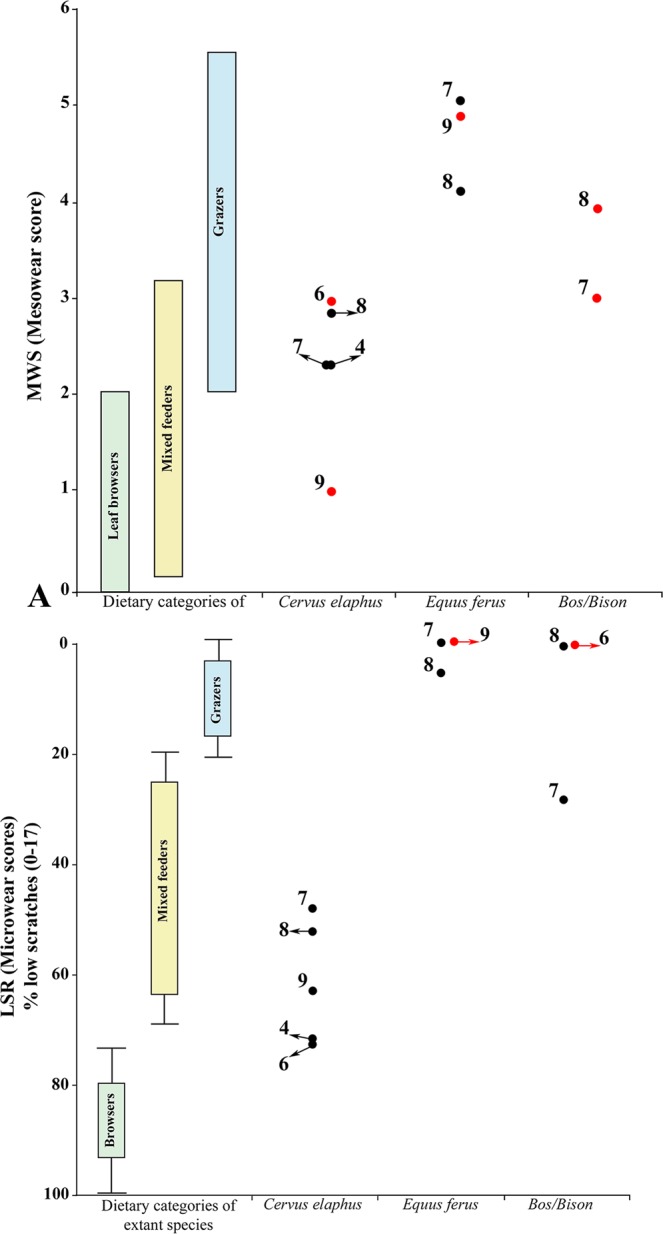
Mesowear (**A**) and microwear (**B**) results of the three selected ungulates taxa obtained from the Covalejos Cave (levels 4, 6, 7, 8 and 9) and dietary bars of extant ungulates^37,45^. Red dots: n < 5 specimens.

Thus, this research investigates the performance of the combined use of dental wear and cementum techniques in investigating the Covalejos Cave (Velo de Piélagos, Cantabria, Spain) (Fig. 1). This archaeological site provides optimal conditions for such a study not only because of its well-defined stratigraphic sequence dated to the Middle and Upper Palaeolithic but also because of its regional location, which is considered a flora and fauna refuge^67–69^. The faunal samples selected for analysis come from the Mousterian levels 4, 6, 7, 8 and 9 (Fig. 2). This chronological context marked by a series of climatic fluctuations on a regional scale also allows us to make observations from the palaeoecological and archaeological perspectives. In this sense, a number of bioarchaeological analyses have already been conducted on this site, including studies on charcoal^70–72^ and pollen remains^73^, micromammal^74^ and macromammal assemblages^75,76^, and recently on isotope analysis of ungulate bones and teeth^77^. However, to date, no extensive study on the feeding patterns and on the ecological niche of ungulates from the Mousterian sequence has been carried out nor have studies on human occupation and mobility patterns been conducted.

Therefore, three main objectives of this study are as follows:

1. To identify the dietary patterns and the ecological niche occupied by each investigated taxon (*C. elaphus*, *E. ferus*, *Bos/Bison*), as well as the prevailing environmental conditions for each Mousterian level (4, 6, 7, 8 and 9) of the Covalejos Cave;
2. To assess the seasonality of prey mortality events and human occupation(s) in the cave in addition to the seasonal scheduling of hunting activities; and
3. To test the accuracy of the combined method involving dental wear and cementum analyses in archaeological contexts.

## Site and Materials

### Sampling site

The Covalejos Cave (Velo de Piélagos, Cantabria, Spain) is located in the western part of the Sierra del Peñajorao, approximately 3 km from the current coastline and approximately 48 m above sea level; it is situated adjacent to the last stretch of the Pas River and near its mouth. It has developed a karst landscape with a north–south orientation, which is a characteristic of the region, and surrounded by calcareous mountains of no more than 250 m high (Fig. 1)^78^. Eduardo de la Pedraja discovered this cave in 1872 and excavated it until 1879. This site was first mentioned by Sanz de Sautuola^79^ in ‘Breves apuntes sobre algunos objetos prehistóricos de la provincia de Santander’ at the end of the nineteenth century followed by several authors in the twentieth century^80–83^^.^ At the end of the twentieth century and in the early twenty-first century, Montes and Sanguino conducted several archaeological campaigns (1997–1999 and 2002) to perform cleaning fieldwork, archaeological surveys and documentation of the stratigraphic sequence of this site.

The archaeological fieldwork revealed a 4-metre deep stratigraphic sequence wherein 15 levels were distinguished (Fig. 2), including 12 archaeological levels from possibly the Acheulean (level 15), the Mousterian (4, 6, 7, 8, 9 and 11) and the early Aurignacian (2 and 3), as well as 1 sterile level (5) and 3 levels formed by stalagmitic crusts (10, 12 and 14) (Fig. 2) (Sanguino and Montes, 2005). Diverse works^78,84^ frame the Mousterian sequence under the isotopic stage (MIS) 5 to 3, yielding a chronology ranging from 101,000 BP (U/Th) for level 13 to 30,380 ± 250 BP (AMS: GrA-22443) for level 2.

The macromammal faunal assemblage mainly consisted of temperate taxa, including *Ursus spelaeus, Crocuta crocuta, Felis silvestris, Canis lupus, Vulpes vulpes, E. ferus, E. hydruntinus, Stephanorhinus* sp., *Sus scrofa, Megaloceros* sp., *C. elaphus, Capreolus capreolus, Dama* sp., *Bos/Bison, Capra pyrenaica, Rupicapra rupicapra* and *Oryctolagus cuniculus*^76^. The area also contains remains of strictly cold-adapted taxa, such as *Rangifer tarandus* (in levels 2, 3, 7, 8 and 9). The micromammal fauna mainly consisted of taxa that are most commonly found in areas with relatively humid and temperate conditions and can thrive in diverse biotopes ranging from open landscapes to forested and shrub areas and water courses, as follows^74^: *Apodemus sylvaticus, Arvicola terrestris*, *Clethrionomys glareolus, Crocidura russula, Erinaceus europaeus, Galemys pyrenaicus, Pliomys lenki, Microtus agrestis, Microtus arvalis, Sorex araneus, Talpa europaea* and *Terricola lusitanicus*. Also included are cold-adapted taxa, such as *Marmota marmota* (in levels 8 and 9), *Microtus oeconomus* (in levels 7, 8 and 9) and *Microcebus* (in levels 2 and 8), as well as taxa adapted to rocky areas (*Chionomys nivalis* in level 8).

Palaeoenvironmental studies based on pollen and charcoal analyses^70,73^ reveal a landscape with predominant open spaces dotted with wooded and shrub areas and marked by a continuous alternation of *Pinus-Betula* and *Quercus* as well as of shrub taxa, such as *Juniperus* and *Ericaceae*, whose proportions changed depending on the relative humidity characterizing each level.

## Material

The material sampled for the meso- and microwear studies comprised 225 teeth belonging to *C. elaphus* (n = 137), *E. ferus* (n = 49) and *Bos/Bison* (n = 39); these teeth were obtained from all of the Mousterian levels of Covalejos Cave. To avoid any bias due to post-mortem damages (see Supplementary Fig. S1D), we screened all teeth with our naked eyes and under a stereomicroscope to identify and eliminate those with taphonomical alterations (see Methods). Out of the 225 initial samples, 84 teeth (*C. elaphus*, n = 59; *E. ferus*, n = 22; *Bos/Bison*, n = 3) were subjected to mesowear analysis and 195 teeth (*C. elaphus*, n = 116; *E. ferus*, n = 43; *Bos/Bison*, n = 36) were subjected to microwear analysis (Table 1). Regarding cementum analysis, we obtained 63 thin sections from 21 teeth of *C. elaphus* and *Bos/Bison*, and these teeth were excavated from levels 4, 6, 7 and 8 (Table 2; see Table S1 for percentages).

**Table 1:** Summary of meso- and microwear data for the Covalejos Cave.

Taxa	Level	Mesowear			Microwear												
		N	MWS	SD	N	LSR	NS	CV*	SD	NP	CV*	SD	%LP	%G	%XS	%ch	SWS
*Cervus elaphus*	4	12	2.3	0.49	29	72.41	16.93	0.1	1.66	26.17	0.18	4.69	89.66	0	48.28	3.45	0.93
	6	2	3	1.41	15	73.33	15.8	0.1	1.6	21.83	0.16	3.34	93.33	0	46.67	0	0.73
	7	24	2.3	1.09	34	47.05	17.35	0.12	1.97	25.71	0.15	3.84	100	14.71	47.06	8.82	0.68
	8	20	2.7	0.93	31	54.83	17.18	0.12	2.06	21.82	0.19	4.03	100	0	25.81	12.9	0.94
	9	1	1	—	8	62.5	17.06	0.14	2.28	23.69	0.10	2.31	100	0	50	25	0.63
*Equus ferus*	4	—	—	—	—	—	—	—	—	—	—	—	—	—	—	—	—
	6	—	—	—	1	—	—	—	—	—	—	—	—	—	—	—	—
	7	7	5.3	0.76	10	0	21.8	0.18	3.77	27.45	-	4.62	100	0	90	20	1
	8	14	4.4	0.84	30	6.67	21.28	0.11	2.27	24.17	0.13	3.14	93.33	0	70	13.33	1.13
	9	1	5	—	2	0	25.25	0.11	2.48	13.5	0.18	2.12	100	0	100	100	1
*Bos/Bison*	4	—	—	—	1	—	—	—	—	—	—	—	—	—	—	—	—
	6	—	—	—	4	0	21.25	0.08	1.66	13.88	-	0.85	-	0	75	100	1
	7	2	3	0	21	28.57	20.38	0.15	3.04	27.24	0.19	5.12	95.24	0	61.9	19.05	1.05
	8	1	4	—	13	0	21.69	0.12	2.44	24.73	0.11	2.77	100	0	76.92	7.69	0.77
	9	—	—	—	—	—	—	—	—	—	—	—	—	—	—	—	—

Abbreviations: N = number of specimens; MWS = mesowear score; LSR = microwear score; NS = average number of scratches; CV* = corrected coefficient of variation; SD = standard deviation; NP = average number of pits; %LP = percentage of individuals with large pits; %G = percentage with gouges; %XS = percentage with cross scratches; %ch = percentage with coarse/hyper-coarse scratches; SWS = scratch texture analysis.

**Table 2:** Summary of cementum data obtained from the Covalejos Cave.

Taxa	Level	Ref.	Tooth	N^o^ CB	LCB	%Dev.	Season of Death
*C. elaphus*	4	CL 1	m2 R	OB + 3 (+TB)	TB	35.75	Middle Good Season
		CL 249	m2 L	13	OB	OB	Bad Season
		CL 262	M1 R	5 (+TB)	TB	23.89	Beginning Good Season
		CL 268	m1 R	7	OB	OB	Bad Season
		CL 275	m3 R	3 (+TB)	TB	38.86	Middle Good Season
	7	CL 97	m3 R	5 (+TB)	TB	10.39	Beginning Good Season
		CL 158	M2 R	9	OB	OB	Bad Season
		CL 197	M2 R	—	—	—	No data
		CL 222	m3 L	4 (+TB)	TB	52.24	Middle Good Season
		CL 225	m2 L	6 (+TB)	TB	19.67	Beginning Good Season
	8	CL 2	m3 R	5	OB	OB	Bad Season
		CL 3	m2 R	8 (+TB)	TB	14.19	Beginning Good Season
		CL 302	m2 R	4	OB	OB	Bad Season
		CL 349	M1 R	9 (+TB) (at least)	TB	28.82	Beginning Good Season
		CL 360	m1 L	5 (+TB)	TB	82.51	End Good Season
*Bos/Bison*	6	CL 112	m2 L	—	—	—	No data
	7	CL 146	m2 L	—	—	—	No data
		CL 327	m3 R	—	—	—	No data
	8	CL 50	M1 R	5 (+TB)	TB	48.78	Middle Good season
		CL 51	M1 L	5 (+TB)	TB	29.85	Beginning Good Season
		CL 291	m1 L	3 (+TB)	TB	92.84	End Good Season

Abbreviations: Ref. = reference number assigned by the authors; N^o^ CB = number of pairs of bands observed in the acellular cementum; LCB = last cementum band observed; %Dev. = % growth ratio of the last cementum increment; Seasonality = season of the individual death; OB = dark and high mineralized (opaque) cementum band; TB = clear and low mineralized (translucid) cementum band; M = upper molar; m = lower molar; L/R: left or right.

## Results

We analysed the dietary patterns of three major ungulate taxa (*C. elaphus*, *E. ferus* and *Bos/Bison*) and the seasonality of Neanderthal hunting activities in all of the Mousterian sequences of the Covalejos Cave (levels 4, 6, 7, 8 and 9).

### Ungulate dietary patterns and environmental conditions

*Tooth mesowear*. *C. elaphus* shows homogenous mesowear values (MWS) throughout the Mousterian sequence (Table 1), ranging from 2.3 to 2.7; this homogeneity was obtained when the values corresponding to only one or two specimens from a unique level (9 and 6, respectively) were excluded. The MWS of the samples from levels 4 and 7 is 2.3, which is the lowest value, whereas that of the samples from level 8 is 2.7. These samples are classified as grass-dominated mixed feeder (Fig. 2A). Levels 6 (MWS = 3) and 9 (MWS = 1) yielded very few samples suitable for analysis (n = 2 and n = 1, respectively; Table 1), so they are simply mentioned but are not taken into account in the interpretations. Data suggest that the red deer from levels 6 and 9 have a grass-dominated mixed feeder diet and a browser diet, respectively (Fig. 2A).

The MWS values for *E. ferus* are only significant for levels 7 and 8 (5.3 and 4.36, respectively; Table 1). These results indicate a grazing diet in both levels (Fig. 2A). The sample from level 9 is represented by a single tooth with an MWS of 5, which falls within the MWS range for levels 7 and 8 (Fig. 2A).

For *Bos/Bison*, only levels 7 (n = 2) and 8 (n = 1) contained a few teeth suitable for a preliminary analysis, with MWS values of 3 and 4, respectively (Table 1). *Bos/Bison* from level 7 suggest a grass-dominated mixed feeder, whereas the sample from level 8 could indicate a pure grazer diet (Fig. 2A).

#### Tooth microwear

**Figure 3:**
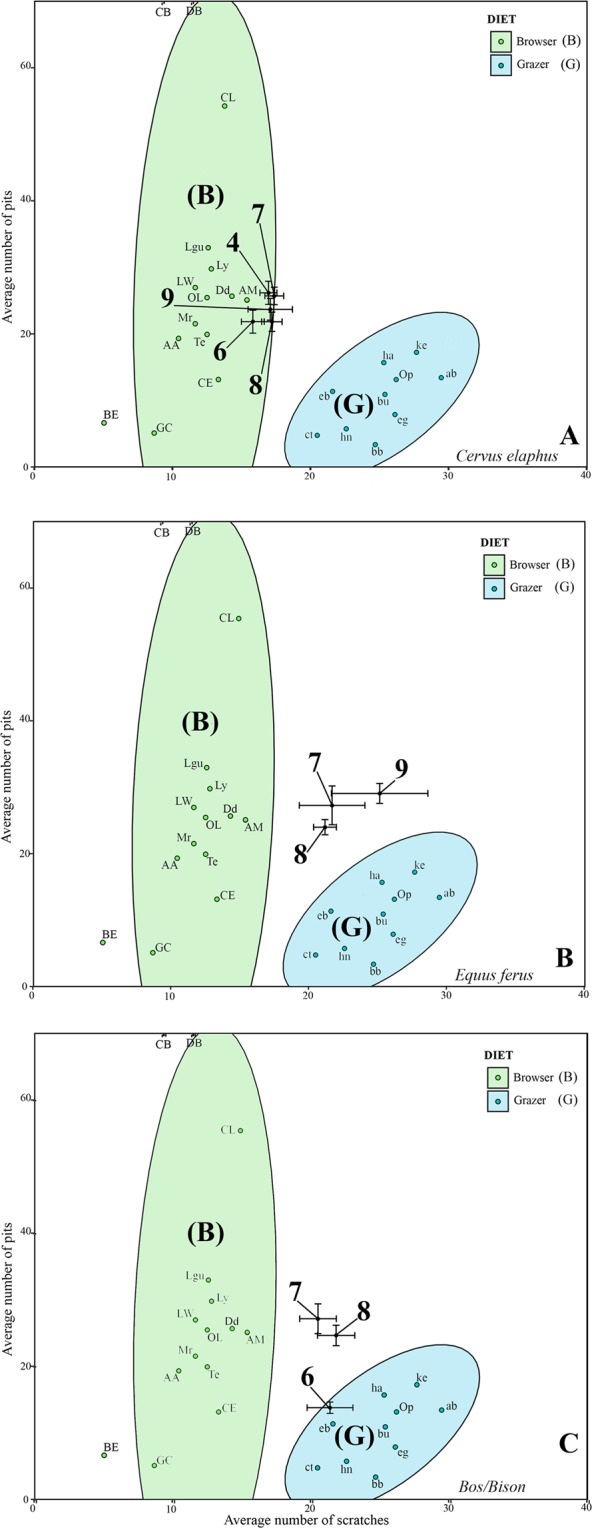
Bivariate plot of the average number of pits and scratches in the three selected ungulates taxa from the Covalejos Cave: *Cervus elaphus* (**A**), *Equus ferus* (**B**), *Bos*/*Bison* (**C**). Error bars correspond to the standard deviation (±1 S.D.) for the fossil samples. Plain ellipses correspond to the Gaussian ellipses (p = 0.95) on the centroid for the extant leaf browsers (**B**) and grazers (G) from Solounias and Semprebon^45,72^. Abbreviations: ab (*Alcelaphus buselaphus*), AA (*Alces alces*), AM (*Antilocapra americana*), BE (*Boocercus eurycerus*), bu (*Bubalus mindorensis*), CB (*Camelus bactrianus*), CL (*Camelus dromedarius*), CE (*Cervus elaphus*), ct (C*onnochaetes taurinus*), Dd (*Dama dama*), DB (*Diceros bicornis*), eg (*Equus grevi*), GC (*Giraffa camelopardalis*), ha (*Hippopotamus amphibius*), hn (*Hippotragus niger*), ke (*Kobus ellipsiprymnus*), Lgu (*Lama guanicoe*), LW (*Litocranius walleri*), Ly (*Loxodonta cyclotis*), Mr (*Muntiacus reevesi*).

The average number of scratches (NS) and pits (NP) for *C. elaphus* slightly varies throughout the Mousterian sequence (Table 1). When plotted, the NS and NP values for all the Mousterian levels (4, 7, 8 and 9) fall within the limits of the confidence interval for modern browsers, except those for level 6, which fall within the confidence ellipse (Fig. 3A). In terms of the low scratch range (LSR) (Fig. 2B), the LSR values of the samples from levels 4 and 6 lie between those of the browser feeders and browse-dominated mixed feeders, confirming the general tendency observed in the bivariate plot (Fig. 3A). Instead, levels 7, 8 and 9 show NS and NP values characteristic of a mixed feeder (Fig. 2B) (Fig. S1A). The scratch width score (SWS) observed throughout the sequence shows the predominance of fine scratches among some coarse scratches (Table 1). This pattern is related to a high level of attrition, which corresponds to browsers and browse-dominated mixed feeders. The high proportion of large pits (LP) observed shows a predominance of browse dietary preferences for almost all individuals from the whole sequence (Table 1) (Fig. S1A), whereas the absence of or low percentage of gouges eliminates the possibility of fruit or seed consumption (Table 1). Cross scratches (XS) are usually related to bark and coarse stem feeding, and the percentages of XS in this study indicate a moderate consumption of such resources, typical of animals with mixed feeder diets wherein they switch between attritive and abrasive food items (Table 1).

Regarding *E. ferus*, the average NS and NP values are only available for levels 7 and 8, whereas only approximate results are available for level 9, because the few teeth available are not suitable for analysis (Table 1). All these levels are unexpectedly located outside the grazer confidence ellipse due to a high proportion of pits, higher than expected for grazer animals (see Supplementary Fig. S1C). As teeth presenting taphonomical alterations were discarded from the studied assemblage (see Supplementary Fig. S1D), we eliminated the possibility of post-mortem alterations to explain the high proportion of pits. Additionally, the percentage of scratches falls within the limits of the grazer confidence interval (x-axis), indicating a dietary pattern similar to that of a typical grazer (Fig. 3B). The LSR for levels 7 and 8 corroborates the grazer diet of horses, as also suggested for level 9 (Fig. 2B). The SWS values corresponding to a mixture of fine and coarse scratches associated with a high XS ratio and the absence of gouges (Table 1) also indicate the preference for a grazer diet.

For *Bos/Bison*, the average NS and NP values available belong to levels 6, 7 and 8, although level 6 yields a small sample size that provides approximate information only (Table 1). Values for levels 7 and 8 plot are close to each other but they lie outside the confidence ellipse for modern grazer (Fig. 2C), as do those for horses (Fig. 2B). However, the LSR values rather present a great variability (Table 1) wherein large bovids from level 7 have a grass–dominated mixed feeder diet (Fig. S1B), whereas those from level 8 have a pure grazer diet (Fig. 2B). Based on the NS and NP values, the LSR for level 6 also indicates a pure grazer diet.

### Seasonal patterns of prey acquisition

#### Tooth microwear

**Figure 4:**
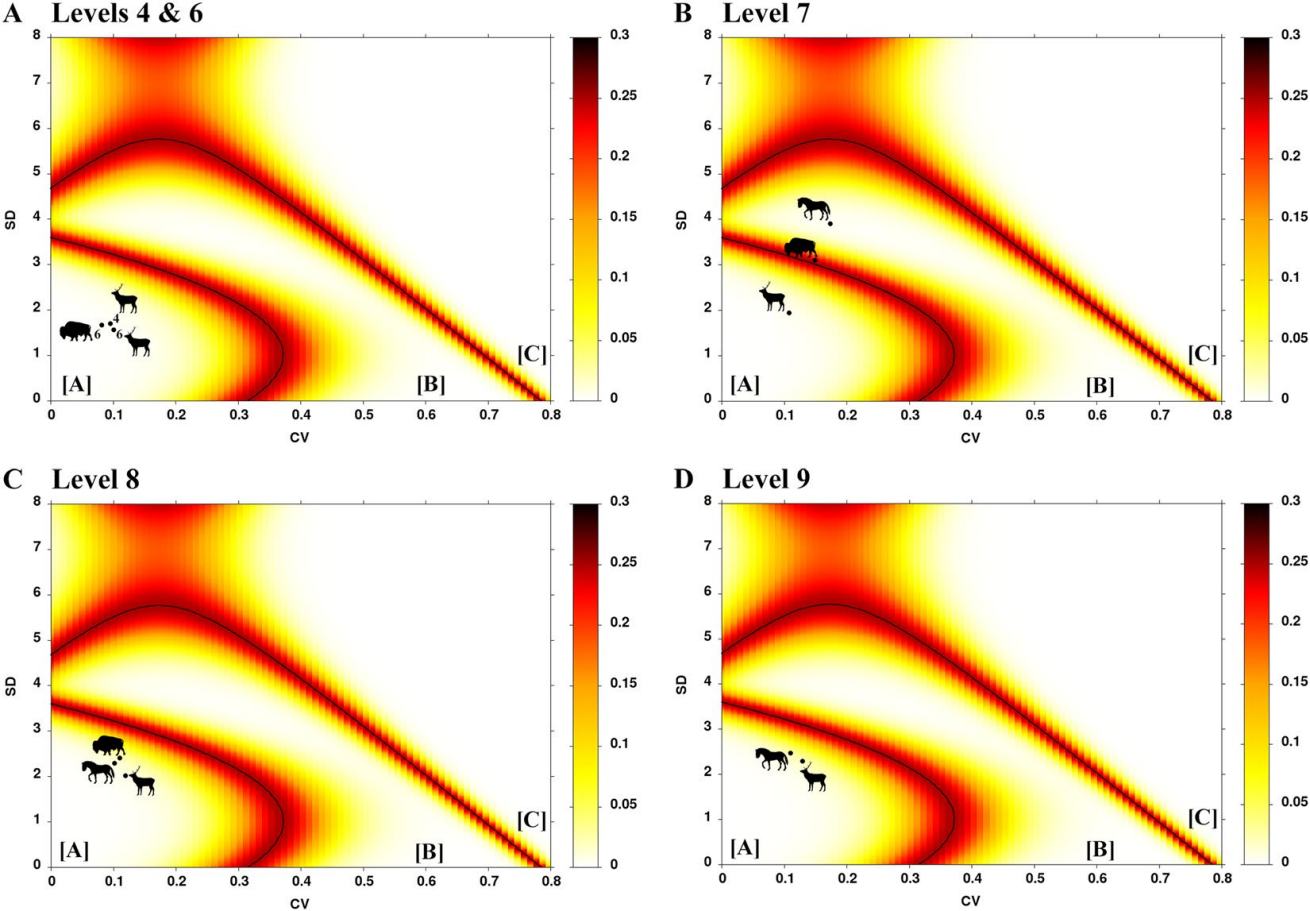
Microwear data for *Cervus elaphus*, *Equus ferus* and *Bos/Bison* from levels 4, 6, 7, 8 and 9 of the Covalejos Cave. Bivariate plot: standard deviation (SD) and coefficient of variation (CV) calculated based on the number of scratches. Boundary lines between the three areas with the error probability (heat map). A = seasonal or shorter events; B = longer than one season; C = at least two separate events that occurred in different non-consecutive seasons. Silhouettes were downloaded from the PhyloPic database (www.phylopic.org) under the Public Domain Dedication 1.0 license (*Cervus elaphus* and *Bos bison*) and the Creative Commons Attribution 3.0 Unported license (*Equus ferus*).

. In levels 4 and 6, the microwear values for *C. elaphus* present a low intraspecific variability (Table 1), with both levels plotting within zone A (Fig. 4A). For *Bos/Bison* (Table 1), the results are limited by the small sample size, but the values also lie within zone A (Fig. 4A). The meso- (MWS) and microwear (LSR) results for red deer show discrepancies within the same dietary group, that is, MWS indicate mixed feeder and LSR reflect browse or browse-dominated mixed feeder (Fig. 2A,B).

In level 7, data available for *C. elaphus, E. ferus* and *Bos/Bison* (Table 1) present a high interspecific variability; red deer plot in zone A and horses in zone B (Fig. 4B). Particularly, *Bos/Bison* plots on the boundary of zones A and B; thus, we cannot identify the duration of the mortality event (Fig. 4B). The MWS and LSR results for red deer do not show discrepancies since both indicate a mixed feeder pattern. Also, discrepancies were observed in the MWS and LSR values for horse as grazer and large bovid as mixed feeder (Fig. 2A,B).

In level 8, the three taxa present a low interspecific variability (Table 1). The bivariate plot shows that altogether, *C. elaphus*, *E. ferus* and *Bos/Bison* fall within zone A (Fig. 4C). The MWS and LSR results for all taxa do not show discrepancies: red deer as mixed feeder and horse and large bovid as grazers (Fig. 2A,B).

Level 9 presents the smallest sample from the Mousterian sequence (Table 1). Red deer plot within zone A, similar to our observations in previous levels (Fig. 4D); *E. ferus* (n = 2) (Table 1) also plots within zone A, but this result is not significant due to the small sample size. Neither the red deer nor the horse MWS or LSR show discrepancies, the former species being a browse-dominated mixed feeder and the latter being grazers (Fig. 2A,B).

#### Dental cementum analysis

The cementum analysis gave positive results since 17 (81%) of the 21 teeth we sampled had clear and measurable seasonal bands (Table 2) (see supplementary Table S3). These samples present dental tissues that are generally well-preserved, that is, most of the main internal tooth tissues were visible. Within the cementum layer, suitable regions of interest could be selected for analysis. Most of the Mousterian levels provided reliable data, except level 6, where the single tooth that could be prepared had a poorly preserved cementum, as well as level 9, where no specimen was available.

For level 4, the sample consists of five *C. elaphus* teeth (NMI = 5) with clear cementum deposits, including the last band (LCB). The results are characterized by a high inter-individual variability: in one case, the LCB is a starting translucid band (TB), two cases are halfway through TB development, and two other teeth present a last annulus (Table 2).

The sample from level 7 contains only five *C. elaphus* teeth with positive results (Table 2). The two *Bos/Bison* teeth (n = 2) and another red deer tooth we sampled and prepared did not show a well-preserved cementum or readable areas of AC. Again, the red deer show a high inter-individual variability in terms of mortality pattern, since the last increments correspond to several seasons of the year. Two individuals show a starting TB, another has a halfway developed TB, and the last one shows an opaque band (OB) (Table 2).

Level 8 contains the largest sample with good cementum preservation. The season of death can be estimated for eight individuals consisting of *C. elaphus* (n = 5) and *Bos/Bison* (n = 3) (Table 2). The results for both taxa exhibit a high inter-individual variability characterized by two distinct seasonal patterns. For the red deer, the analysis of thin sections reveals one individual with an ending TB, two others with an OB and the remaining ones with a starting TB.

## Discussion

### Ungulate dietary spectrum and palaeoecological inferences

The three ungulate taxa studied in Covalejos Cave show a wide spectrum of diets, ranging from mixed feeders (browse- and grass-dominated) to pure grazers. This finding highlights the availability of a wide range of resources, supporting the hypothesis that the ungulates from the Covalejos Cave hardly changed their dietary traits throughout the Mousterian sequence.

Certainly, the palaeodiets of the fossil ungulates could be inferred by comparing their average NS and NP values with those of the extant ungulates with known diets. The results are considerably useful when a sample plots within the confidence ellipses for browsing or grazing diets. However, given that an intermediate group exists, that is, mixed feeders, the NS and NP values should be compared with the meso- (MWS) and microwear (LSR) data in order to reach accurate interpretations.

The extant *C. elaphus* is a mixed feeder that seasonally varies its diet from browsing to grazing^45,85–87^. According to the mesowear (MWS), the abrasive properties of the food consumed all year round by red deer as observed in the Mousterian sequence of the Covalejos Cave are strongly correlated with the grass-dominated mixed feeder diet. Moreover, daily/weekly (short temporal) dietary traits highlighted by microwear (LSR) present low intra-site variations among the mixed feeders as shown by the NS/NP confidence ellipse (Figs 2A,B and 3A). However, these variations are not sufficiently strong to drastically modify the diet or to alter the ecological niche of red deer, which periodically changes its diet from attritive to abrasive resources and vice versa. This alternating consumption is also observed in terms of additional microwear features, such as regular occurrence of large pits, gouges, XS and scratch texture, which indicate a mixed feeder dietary pattern.

*E. ferus* is a typical grazer that normally feeds on large amounts of grasses^88–90^. The studied horse samples at the Covalejos Cave consistently showed a pure grass-feeding pattern and a similar ecological niche throughout the sequence (Fig. 2A,B). This finding is confirmed by additional dietary observations (i.e. %XS and %G). However, the samples are characterized by an exceptionally high frequency of pits, which plot outside the typical grazer dietary range (Fig. 3B; Table 1). Such evidence could be initially attributed to the consumption of attritive elements (e.g. leaves) and hard elements (e.g. twigs). Likewise, the intake of seeds or fruits produce a greater frequency of large pits, puncture pits and gouges^45^. Nevertheless, except for the high pit rate, the dietary evidences recorded in horse do not support any attritive resource consumption since all lines of evidence are indicative of strict consumption of abrasive resources (Fig. 2A,B; Table 1)^45,91^. Even so, lines of evidence reveal an unexpected high rate of pits and large pits associated with browser feeding in arid contexts^92^. Considering this evidence, we suggest that the horse dietary signal (i.e. high number of pits) could also be affected by high proportions of dust, grit or/and other extrinsic particles^92,93^ (post-mortem alterations previously disregarded).

*Bos/Bison* does not provide sufficient data to infer its average annual diet through mesowear analysis (Fig. 2A; Table 1) even though the microwear plot generally indicates a mixed feeder dietary pattern (Fig. 3C). However, the short temporal scale recorded by microwear suggests an alternating feeding patterns of pure grazing (levels 6 and 8) and grass-dominated mixed feeding (level 7) (Fig. 2B). Similar to the horse (Fig. 3B), this taxon presents a high proportion of pits in levels 8 and 7 (Fig. 3C), and this finding can be explained by a significant occurrence of dust and/or extrinsic particles in their food resources, resulting in a shifted dietary pattern. However, *Bos/Bison* slightly shifts its annual feeding pattern from consuming highly abrasive resources (level 8) to consuming resources with moderate abrasiveness (level 7) as also reflected by its daily/weekly diet (from pure grazing to grass-dominated mixed feeding) (Fig. 2A,B). Additional microwear features, such as the reduction of the XS rate, is also indicative of reduced abrasive resource consumption (Table 1). Large bovids seem to slightly vary their ecological niches from level 8 to 7 (Fig. 2A,B); however, with the lack of mesowear data, we can propose alternative hypotheses. Another possible interpretation is related to the difficulty in identifying whether the investigated large bovids belong to the same taxa or not. *Bos primigenius* and *Bison priscus* present dietary plasticity but with different general dietary preferences, mainly grazer versus more mixed feeder diets, respectively^85,94–98^. The observed differences between levels 8 and 7 could also be due to the presence of both *B. primigenius* and *B. priscus* or even to the variations in the proportion of these two species at different levels.

The three ungulates studied show diverse dietary patterns (i.e. from pure grazer to mixed feeder, including those with both browse and grass preferences), wherein each taxon occupies a different ecological niche, suggesting the absence of interspecific competition. However, in contexts where there is an absence of or low incidence of environmental changes, such as climate refuges, dental wear interpretations could be biased by the lack of variability (also by low variability) in the available resources. This perspective raises the question of (1) whether our data represent methodological issues related to the resolution of the two dental wear techniques due to particular environmental conditions or (2) whether the consequence of mortality events occurring over long periods of time that result in the averaging of diverse dietary traits vary throughout the year.

The three main ungulate taxa had different diets that altogether cover a wide dietary spectrum. Moreover, they are constant over time and suggest the presence of several habitats surrounding the cave as can be seen in the Mousterian sequence. Quantitative proportions of each species (using the minimum number of individuals) are taken into account for the palaeoecological interpretations^69^. In fact, *C. elaphus* is the dominant taxa in all of the studied Mousterian levels of the Covalejos Cave, whereas the proportions of *E. ferus* and *Bos/Bison* were oscillating^76^.

The smallest tooth assemblage in the Mousterian sequence is recorded at level 9, and it is dominated by *C. elaphus*. The dietary traits of red deer suggest the availability of wooded areas along with open spaces around the cave since the animals fed on leaves and shrubs (attritive) as well as on grasses (abrasive), although they show preferences for attritive resources (Figs 2A,B; 3A). Furthermore, the presence of *C. capreolus* (woodland species) and the marginal presence of *E. ferus* (open spaces) supports the view of a patched landscape with predominance of woodlands over open spaces under relatively temperate and humid conditions. However, the occurrence of *R. tarandus* and *M. oeconomus* could be considered an indicator of colder environmental conditions.

In level 8, *C. elaphus* and *E. ferus* are the dominant taxa followed by *Bos/Bison*. Horses and large bovids are largely represented in level 8 relative to that in level 9, and their diet consists of highly abrasive resources, suggesting the expansion of the grasslands in level 8 compared with that in level 9. The consumption of abrasive resources by red deer slightly increased, but they are still within the mixed feeder group, indicating the presence of wooded areas around the site^70,74,76^. The predominance of abrasive diets and the high numbers of pits observed in the three taxa suggest an intensification of aridity, possibly as a consequence of a cold climatic pulse. This finding is supported by the presence of *R. tarandus*, *M. oeconomus* and *M. marmota* in the assemblage^74^. Otherwise, the record of *Glis glis* and *Clethrionomys* (i.e. temperate taxa), along with the alternation of *Pinus-Betula* and temperate taxa, such as *Quercus robur* and *Castanea sativa*, indicates the maintenance of relatively humid conditions and wooded areas even during cold and dry pulses^70,74^.

The dietary traits observed in level 7 suggest an environmental trend similar to that in level 8, wherein the seasonal intake of abrasive resources by *C. elaphus* has increased, and *E. ferus* shows the highest abrasive values in the sequence (Fig. 2A,B). However, the proportion of horses is decreasing and that of *Bos/Bison* is increasing relative to that in level 8. In addition, the diet of large bovids shifts towards reduced consumption of abrasive resources, which is possibly related to the different proportions of the two large bovid species (*B. primigenius* and *B. priscus*), which have different dietary habits, and not to their ecological competition with horses. Moreover, the three main ungulates maintain high numbers of pit, suggesting that aridity must still be intense at the time. The ungulate dietary traits suggest a landscape that is mainly dominated by open areas as well as by the presence of some wooded areas as indicated by the alternation of *Pinus–Betula*^70^. Certainly, the anthracological record show similar features than level 8, indicating relatively humid and temperate conditions^70^. Cold environmental conditions must still be present since *R. tarandus* and *M. oeconomus* were recorded, and the modification of the dietary pattern of the large bovids, along with the appearance of another temperate micromammal taxa (i.e. *Microtus agrestis*)^74^, could suggest the beginning of a slight improvement of the environmental condition.

In levels 6 and 4, the arid trend seems to reverse or at least not increase. However, *C. elaphus*, the dominant taxon in both levels, show annual dietary trends similar to those in levels 8 and 7, and the daily/weekly diet indicates a seasonal tendency, with an increment for attritive resources even higher than that in level 9 (Fig. 2A,B). *E. ferus* and *Bos/Bison* are marginally represented in levels 6, and level 4 did not yield any horse teeth. The low frequency (level 6) or absence (level 4) of horse could be interpreted as a reduction in open spaces as horses generally require extensive grass plains^76^. However, the abrasive diet of the marginally represented large bovids^76^ suggest the presence of open spaces around the site. Considering the dietary traits observed in levels 6 and 4, we suggest the occurrence of a reforestation at the expense of open landscapes for a possible recovery of relative humidity. In fact, an increase in the number of temperate-adapted micromammal taxa, such as *Microtus agrestis* and *Plyomis lenki*, has been recorded^74^. Finally, the *Pinus–Betula* alternation, indicating temperate conditions, is a constant in the Covalejos sequence^70^.

The mosaic landscape as suggested by dental wear is in concordance with other ecological findings discussed above^70–74^^,84^, wherein cold and dry pulsations do not influence the preeminent humid and temperate characteristics of the environment. Jones *et al*.^77^ suggested that climatic oscillations are not sufficiently intense to produce strong modifications in the surrounding vegetation cover. Such environmental stability preserved the relative abundance of resources around the cave throughout the year, allowing the ungulates to feed on a broad spectrum of plant resources. Each taxon occupies a different ecological niche, suggesting the absence of interspecific competition among different taxa. Indeed, temperate environments present slight seasonal variations that could affect the surrounding vegetation assemblage, but certain types of browse (trees, bushes or herbaceous plants) must have been perpetually available. This seems to be the case for Covalejos Cave, wherein red deer have primarily consumed foods with similar attritive–abrasive properties no matter the season and resources available. In fact, the attritive–abrasive values of some types of vegetal resources could have also varied over the seasons^45^, suggesting that the correlation between annual and daily/weekly dietary patterns in the Mousterian sequence imply that the surrounding vegetation had not undergone significant variations.

In addition, the absence of strong discrepancies between meso- and microwear results in the Mousterian sequence, that is, between diets on long- and short-term scales^51^, indicates a long mortality pattern for the three investigated ungulate taxa. The question is whether the ungulates’ dietary stability and mortality patterns based on dental wear really correspond to long-term death events or whether they correspond to a biased set of information that is being affected by low environmental variability in the context of climate refuge.

**Figure 5:**
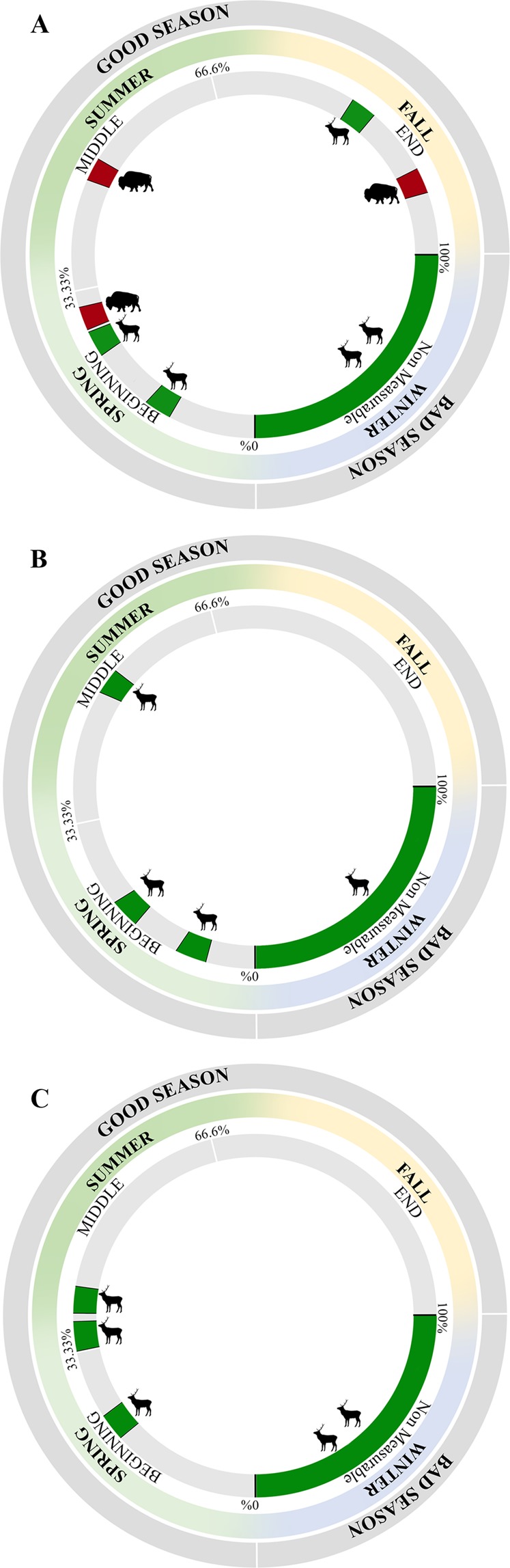
Schematic representation of hunting events for *Cervus elaphus* (green) and *Bos/Bison* (red) in the Covalejos Cave. Percentages correspond to the degree of development of the last translucent cementum band (TB). (**A**) level 8; (**B**) level 7; (**C**) level 4. Silhouettes were downloaded from PhyloPic database (www.phylopic.org) under the Public Domain Dedication 1.0 license (*Cervus elaphus* and *Bos bison*).

More specifically, combining these results with the findings of dental cementum analysis offer more accurate interpretations on the ungulate dietary behaviour and mortality patterns in Covalejos Cave (Table 2). The killing events among *C. elaphus* took place in different seasons within the annual cycle in levels 8, 7 and 4, as well as among *Bos/Bison* in level 8 (Fig. 5A,C). The seasonality of deaths in both taxa in environments under expected seasonal climatic fluctuations (i.e. temperate climate), along with the observed low ungulate dietary variability, illustrate a landscape with stable resources all year long. That is, seasonal and global environmental fluctuations throughout the sequence were not sufficiently strong to drive the ungulates to shift their feeding traits and ecological niches. For example, red deer feed on attritive–abrasive resources no matter the season (Fig. 2A,B; Table 1). Indeed, the abundance of resources in the Cantabrian area even during the coldest periods seems to be recurrent, similar to that described by Rivals and Álvarez-Lao^99^ regarding the palaeontological faunal assemblages in the Jou Puerta and Rexidora Caves (Asturias, Spain). Moreover, the combined use of the dental wear and cementum results in this study increases the accuracy of the identification of potential changes in the ecological niche of red deer over time, since the animals were hunted at different times of the year and have maintained the same dietary traits.

### Seasonality and duration of neanderthal occupations

Most of the animal procurement episodes by Neanderthal groups in Covalejos Cave based on the variability of coefficient of variation (CV) seem to correspond to short-term events (levels 4, 6, 8 and 9) (Fig. 4). That is, Neanderthals seem to have occupied the cave in specific moments of similar ecological conditions over time. These data, however, contradict the previous dietary interpretations derived from the comparison of meso- and microwear (MWS and LSR), which indicates long-term or several short-term ungulate mortality events throughout different seasons of the year (Fig. 2A,B)^51^. We are thus facing a situation wherein two possible but contradicting interpretations may be derived from the same data. In fact, considering that microwear is sensitive to resource changes over different seasons^11,45^, one might expect a high microwear variability (i.e. CV/SD) in the absence of discrepancies between MWS and LSR. By contrast, microwear data by itself (CV/SD) show low variability for most of the dietary signals that would indicate similar environmental conditions for the hunting episodes (i.e. short/seasonal mortality event) (Fig. 4A,D). Given this circumstance, the combined use of dental wear and dental cementum results constitute very useful proxies to increase the resolution of information on the Neanderthal occupational patterns because the combined methods are expected to identify seasonality even in the absence of or in low seasonal dietary variability in stable environmental contexts.

Level 8 offers an interesting perspective since cementum and microwear data reveal important results regarding the Neanderthal occupational patterns. The microwear variability (CV/SD) for the three ungulate taxa clearly indicates short-term mortality events (Fig. 4C). Contrarily, cementum analysis results on red deer and large bovid highlight several hunting events in the annual cycle (Fig. 5A), and this finding is supported by consistent dietary patterns (MWS and LSR). Indeed, two different procurement strategies are highlighted in the annual cycle: red deer acquisition took place from fall to spring, and there is no evidence of hunting events for large bovid during winter (i.e. from spring to fall) (Fig. 5A). Therefore, Neanderthal groups performed long-term or several short-term occupations within the annual cycle.

Based on the microwear data, level 7 presents two different prey acquisition events (Fig. 4B). Red deer and large bovids show short-term mortality events, whereas horses show long-term hunting events (Fig. 4B), as also suggested by the MWS and LSR for horse (Fig. 2A,B). The case for the horse turns out to be peculiar as the food for horse is not expected to vary seasonally, as horses usually feed on grasses throughout the year^89,90,100^. However, the observed microwear variability could be attributed to the inter-individual variability in scratches (NS); a consequence of the variation in grass abrasiveness throughout the annual cycle due to plant phenology or to shifts in the amount of grit. Therefore, the cementum analysis result for red deer is consistent with the horse microwear interpretations as they both indicate several seasonal mortality events: from winter to mid-summer (i.e. from bad to mid-good season) (Fig. 5B). The Neanderthal occupation(s) could have taken place either as a long-term episode or as a succession of short-term episodes throughout the annual cycle.

Level 4 shows a pattern similar to that seen in level 7. Red deer microwear data indicate low variability, suggesting short-term mortality events (Fig. 4A); the cementum data indicate that the hunting events took place during the bad season until the first half of the good season (Fig. 5C). In that case, Neanderthal groups preferentially occupied the cave during winter and spring, possibly during the breeding period of red deer, as indicated by the presence of juvenile individuals^76^.

Prey acquisition and occupational patterns in levels 6 and 9 were uniquely approached from dental wear because cementum data are lacking. Microwear data from both levels suggest a unique short-term occupation or several short-term occupations during the seasons with similar environmental conditions; probably, occupations recur approximately during the same season (Fig. 4A,D). However, the absence of discrepancies between ungulate dietary preferences (i.e. MWS and LSR) (Fig. 2A,B), along with the results observed for the previous levels, suggest that long-term procurement activities throughout the year cannot be excluded.

The global occupational pattern inferred for Neanderthal groups in Covalejos Cave is therefore characterized as either long-term occupations or as a succession of short-term occupations throughout the year. This fact would correspond to the general hypothesis that Neanderthal settlement behaviour was characterized by short/seasonal episodes of high mobility within a territory^23,27,30,31^. It is important to take into account that repeated short-term occupations in the annual cycle (i.e. through several seasons) produce similar dental wear values compared with long-term occupations. Both temporal patterns could be distinguished by comparing them using other lines of archaeological evidence. The most likely occupational pattern that occurred in Covalejos Cave would correspond to successive short-term events along the annual cycle as suggested by the occurrence of occasional water presence and carnivore occupations^76^.

The occupational patterns identified in Covalejos Cave are also recognised in several sites in nearby areas^101^. For instance, faunal and lithic studies have identified a residential occupational pattern in the lower part of the sequence of Axlor Cave (levels VI, VII, VIII and M-N)^102^ as well as a wide temporal spectrum of hunting activities in the sedimentary sequence 3 (SQ3) of Arlanpe Cave (Lemoa, Biscay, Spain)^103^. In our study, we recognised similitudes with a previous cementum analysis of the Cantabrian region performed by Pike-Tay *et al*.^101^. Their results suggest a recurring occupational pattern in the Mousterian levels of El Castillo Cave (Puente Viesgo) from late fall/winter to spring/early summer, similar to that observed in Covalejos Cave (levels 8, 7 and 4). On the contrary, the Mousterian levels in Cueva Morín (Villaescusa) and El Pendo (Escobedo) Caves indicate more restricted occupational patterns framed mainly from late fall to winter^101^.

The recurrent presence of Neanderthals in the area could be explained by the refuge conditions offered by the Cantabrian region and by the favourable environmental context, as can be seen from the abundance and diversity of biotic resources around the cave based on pollen and faunal records^73,76^. Thus, our results, along with zooarchaeological data from Covalejos Cave^76^, suggest the role of a base camp or a main hunting camp in the acquisition of local red deer and other ungulates^76^. Indeed, the available data suggest that the procurement activities focused primarily on red deer for most of the year (levels 8, 7 and 4), wherein large bovids were hunted during the good season (level 8) (Fig. 5A–C). These prey procurement patterns suggest that throughout the annual cycle, several short-term occupations have occurred with a focus on targeting selected preys depending on their seasonal availability, as is also suggested for Pech-de-l’Aze’ I^104^.

## Conclusion

A high-resolution technique, such as dental wear analysis, is a useful tool in palaeoecological and archaeological reconstructions and in deepening our knowledge on the palaeoenvironment as well as on the behavioural patterns of human groups that had inhabited it. However, these proxies occasionally do not reach a sufficiently high degree of resolution to arrive at conclusive results. In such a situation, it is necessary to compare the results with data provided by other proxies to obtain more accurate findings. The present research demonstrates that through the combined use of dental wear and dental cementum, detection of potential biases due to the lack of variability in environmental contexts with relative homogeneity is possible when interpreting results given that these techniques provide specific complementary information obtained from different temporal scales. Specifically, the integration of both approaches suggests that the lack of discrepancy between dental wear (meso- and microwear) results does not necessarily indicate a long-term or a succession of short-term access of human groups to ungulate preys. Environmental areas of great stability, such as climate refuges, could conceal relevant information that could alter our interpretations about settlement occupations. This consideration assumes that the results of dental wear should be complemented by palaeoenvironmental studies prior to making archaeological interpretation in order to identify the possible causes of presence/absence of variation in dental wear patterns. In this sense, dental cementum analysis is an efficient complementary proxy as it improves the estimation of the duration of human occupations.

The Cantabrian region seems to encompass prehistoric sites that have played major roles as base/residential camps that were frequently occupied by Neanderthal groups, who were highly mobile throughout the area; moreover, this region covers other sites of seasonal interest, that is, places where these groups performed short-term occupations in search of specific resources. This findings does not imply that these patterns remained unchanged throughout the Mousterian period as seen in the sequences in the Covalejos or Arlanpe caves^103^. The dietary and palaeoecological interpretations derived from the present work suggest that ungulates did not modify their ecological niches because environmental changes were not sufficiently remarkable to have drastically modified the main surrounding vegetation beyond seasonal alternations. The fact that Neanderthal groups did modify their occupational patterns when ungulates did not vary their ecological niches could indicate that the occupational patterns responded not only to the prey behaviour but also to social and cultural factors.

### Methods

#### Tooth mesowear

Tooth mesowear analysis^37^ describes the relative amount of attritive–abrasive dental wear produced by diet within an individual’s lifetime in a relative long temporal scale of months or years. This analysis relies on the macroscopic observation of the sharpness and relief of tooth cuspids, wherein dietary (phytoliths) and non-dietary (dirt, dust and grit) abrasive items present in the resources consumed by the ungulates can modify the morphology of the buccal apices. Because various types of diet cause varying degrees of attrition and abrasion, the resulting cusp morphology can be categorized in relation to the three main herbivore feeding groups, as follows: Browsers (feeding on shrubs and leaves) present low abrasion and high attrition, and they display higher relief and sharper apices than grazers. Grazers (feeding on grasses) show high abrasion and low attrition with lower relief and more rounded or blunt apices. Mixed feeders present intermediate abrasion–attrition values, which create different relief patterns according to the feeding preferences of an individual (e.g. grass or leaf items).

Mesowear variables were originally scored by Fortelius and Solounias^37^ in extant and extinct ungulate species to develop the tooth mesowear analysis, which was subsequently improved by Mihlbachler and collaborators^90^. The new standardized method distinguishes seven scoring stages based on the shape and relief of cusp apices, that is, the mesowear score (MWS), which range from stage 0 (high relief and sharp apices) to stage 6 (no relief and completely blunt apices).

The protocol on tooth selection takes into account the different premises intended to avoid biases in the analysis of a material. The main criteria focus on the optimal preservation of teeth, that is, the crown and the occlusal surface should not present fractures or any kind of damage^37,105^. Tooth age also plays an important role: teeth that do not show apparent wear or those that are extremely worn are discarded. Consequently, due to the strong sensitivity of tooth sharpness relief to ontogenetic age in young individuals (without substantial wear facets) and in dentally senescent individuals (high wear facets), only adult (permanent) teeth with wear facets were selected^106^.

#### Tooth microwear

The tooth microwear method we used in this study was developed by Solounias and Semprebon^45^. This technique describes the set of microscopic features produced on the tooth occlusal enamel surface during mastication by the abrasive particles present in food (i.e. phytoliths, dust and sand adhering to plants)^45^. This method provides information that covers a short temporal scale (i.e. hours/days); the significance of this method is seen in a process known as the ‘last supper effect’^52^, wherein for each new food intake of an individual, a rapid overprinting of a previous set of marks occurs. Therefore, the dental microwear pattern reveals the diet of an individual at the time of death and consequently the local and seasonal environmental resources available shortly before its death^45^.

From among the wear signals, two main types of features can be characterized: scratches and pits. Scratches are elongated features produced by the longitudinal displacement of the jaws during the masticatory process, and they are categorised based on their thickness and depth, as follows: fine (narrow, shallow and with low light refractivity), coarse (wide, relatively deep and with high light refractivity) and hyper-coarse (considerably wide and deep and with low light refractivity). By contrast, pits are circular features produced by the compressive stress in the jaws, and they are classified based on their diameter and depth, as follows: small (regular edge, shallow and with high light refractivity) and large (less regular edges, deep and with low light refractivity)^45,107^.

Thus, a relationship exists between diet and the pattern of dental microwear^45,108^. Depending on the abrasiveness of the resources consumed by the ungulates, dietary groups have been classified as follows: browsers (i.e. those that consume leaves, shrubs and woody plants and have a low NS), grazers (i.e. those that consume pastures and grass and display a high NS) and mixed feeders (i.e. those with wear patterns that are an overlap of the two other patterns) (Fig. 3A–C). The number of pits (NP) in extant browsers and grazers considerably overlap. Qualitative parameters are also scored to better define these dietary groups and to establish subcategories^45,107^. For instance, scratch thickness represents the abrasive properties of food consumed by a population and is measured using the following scoring system (SWS): 0 = fine, 1 = mixed fine-coarse, 2 = coarse, 3 = hyper-coarse and 4 = mixed coarse/hyper-coarse). This scale varies according to sizes of the phytolith and exogenous particles consumed. The percentage of individuals with high abrasive diets (%ch) is calculated by identifying the presence of coarse/hyper-coarse scratches on the tooth enamel surface. The frequency of cross scratches (%XS) refers to the presence of scratches with different directions relative to the main orientation. Large pits (LP) are double-sized pits that indicate bark and branch consumption. Gouges (G) are depressions of irregular morphology and are caused by the consumption of seeds and fruits^45,91^.

To sample an archaeological material, we followed the protocol developed by Solounias and Semprebon^45^. The selected lower and upper molars present a good preservation of the occlusal surface, excluding unworn or heavily worn teeth (e.g. those of juvenile or senile individuals, respectively). In our microscopic observation, we excluded those teeth with taphonomic alterations (e.g. digested, abraded or eroded) that modify or obliterate the microwear signal^109–113^. The enamel surface was cleaned with acetone and 96% ethanol to eliminate any particle or coating. Then a high-resolution silicone (vinylpolysiloxane) was used to mould the dental surface; a low-resolution silicone was used to form the negative mould. Finally, a transparent epoxy was used to create the cast. The microscopic features were optically quantified using a Zeiss-Stemi 2000C stereomicroscope at × 35 magnification with an ocular reticule delimiting a 0.16 mm^2^ square area.

We used two different parameters to infer the feeding patterns of the analysed ungulate taxa: the average NS and NP and the microwear values (LSR).

The average NS and NP combined in a bivariate plot provide information about the ungulate diets. The data obtained were compared with those for modern browsers and grazers^45^ represented on a bivariate plot by their respective 95% confidence ellipses^114^.

We subsequently calculated the percentage of individuals per taxon that show scratch numbers ranging from 0 to 17, that is, the low scratch range (LSR), to distinguish the three main dietary categories^45,46,92^. Browsers present an LSR of 72.73%–100%, whereas grazers show an LSR of 22.2%–0%. The LSR of mixed feeders lies between that of the two other groups (i.e. 20.93%–70%). The last group is an intermediate group which could daily, seasonally or regionally vary their scratch range and fall into the browser or grazer limits depending on the amount of attritive/abrasive items consumed^45,91,92^. Percentages that are close to the browsing or grazing percentages correspond to the browse- or grass-dominated mixed feeding. Note that the borderline percentage between dietary groups is only an approximation since it depends on the modern ungulate populations used as references, so a specific limit value does not necessarily imply a radical change in the diet of an individual.

The duration of prey mortality events, from which we could estimate the temporality of human occupations, was estimated according to the method developed by Rivals and colleagues^115^. They graphically assessed the prey mortality events by combining the coefficient of variation (CV) and standard deviation (SD) obtained from an individual’s scratch variability. They have identified three zones, which reflect the duration of these events. Zone A represents seasonal or shorter events; zone B represents longer period (e.g. more than one season); and zone C indicates at least two separate events in non-consecutive periods. We also take into account the suggestion of Sánchez-Hernández^51^, that is, the discrepancy between meso- and microwear results is indicative of seasonality in prey procurement and ultimately in the occupational pattern.

#### Cementochronology

Cementum analysis, or cementochronology, is a technique that investigates the predictable and seasonal patterns of dental cementum tissue growth, which takes place continuously throughout the tooth life; this technique aims to assess the age and/or season of death of individuals for ecological, zooarchaeological or forensic studies^40,41,54,55,101,116–119^. Cementum is generally located on the root surface, covering the dentine and also part of the enamel surface in some hypsodont taxa (e.g. bovids and equids)^120,121^.

In this study, we focused on the acellular cementum (AC), which is typically located in the middle and upper part of the root and extends up to the junction between the dentin and the enamel tissues. During the appositional annual growth of the AC, two types of increments can be distinguished depending on the rate and degree of mineralization and on the optical properties^40,54,60–62,121^: the translucid band (TB) and the opaque band (OB)^48,54,56,57,63,64,116,117,122^. The TB, also known as the ‘growth zone’^58,65^, is a clear, hypo-mineralized wide band with a fast growth pattern and is generally deposited during seasons of optimal environmental conditions (i.e. the ‘good season’). By contrast, the OB is a hyper-mineralized thin band with a dark appearance, also termed as *annulus* or ‘rest line’ by some authors^54,57,58,65^; it forms during the physiological period of slow growth, that is, the ‘bad season’, such as winter.

Because cementum growth follows a regular annual cycle and because the increments are evenly deposited within the AC, the age and season at death of an individual can be assessed by examining the alternating layers, their nature and their growth rate. Age was inferred by counting the total number of increment pairs and by adding the age of tooth pre-eruption, whereas the season was assessed according to the nature of the last increment (i.e. the TB or the OB). Regarding the latter assessment, when the last increment is a TB, a more precise estimation about the timing of the season (early, middle or late ‘good season’) can be made by calculating the growth ratio of the last increment of the band and compared with the mean thickness of the older increments. Such precision can hardly be reached with the OB since it is generally very thin and difficult to measure when it lies at the outer edge of the cementum layer. According to the data collected by several authors from different modern red deer populations, the time of OB deposition in *C. elaphus* varies from one region to another. However, this variation seems to be linked with latitude, that is, from January to March–April in northern Europe^40,55,123^ (Norway, Scotland and Denmark) and from November to January in southern Spain^33^ (Sierra Morena). Considering these observations, we could postulate a time for OB deposition in Cantabria, that is, approximately from December to February (or a little earlier), but this hypothesis is based on an actualistic approach, and we cannot assert that the same pattern was true in the Middle Palaeolithic times. Thus, we prefer to use herein such general categories as ‘bad season’ or ‘good season’, ‘winter’, ‘spring’, ‘summer’ and ‘fall’. A good season comprises three periods characterized by TB growth percentages, allowing a more accurate estimation of the season of death^40^, as follows: ‘beginning’ (1%–33.3%), ‘middle’ (33.4%–66.6%) and ‘end’ (66.7%–100%).

The selection process starts with a macroscopic observation, wherein teeth presenting cracks and detachment of the cementum tissue within the root, as well as concretion, erosion or manganese deposition, were excluded. Subsequently, a microscopic screening of thin sections was performed to assess the preservation state of the internal tooth tissues (i.e. cementum, dentine tubules, Tome’s granular layer and hyaline layer) and to verify the absence of taphonomic damage (weathering or recrystallization, among others). Technical defects in the thin sections (e.g. watermarks, glue, high thickness of tissues or orthogonality) were also used as criteria for teeth exclusion^40,41,60,64,124,125^. The preparation procedure of tooth samples follows the standard techniques for ground thin sections applied in archaeology contexts^38–41,48–50,60,124^. Upper and lower molars were selected for analysis. As suggested by one of us (LG), we modified the sampling protocol by extracting only one root from each molar to optimize the preservation of tooth integrity. The first step consists of strengthening the root structure by using a transparent epoxy resin to avoid fractures or loss of material and of possible fractures during cutting; such fractures may hinder or prevent the analysis. Next, once the selected root was separated from the crown by using an automatic high-precision saw equipped with a diamond blade disk, the same cutting process was executed to obtain thin sections (0.5–1 mm). The fragility of the thin sections requires a transparent glass support on which they were adhered. To speed up the drying process, we deposited the thin sections in a heater that distributes artificial heat uniformly and constantly for 2 hours and then the sections were allowed to sit for 12 hours for gradual cooling. Finally, the sections were further thinned out by using a manual grinder to allow transmission of light, revealing the dental tissues^40,41,60^.

The cementum banding pattern and other histological features were assessed by examining the thin sections under a polarizing light microscope (Leica 2500 P) at ×10, ×20, ×40 and ×50 magnifications; the microscope was connected to a computer via a high-resolution digital camera (full-screen video). The incremental bands were counted, and the last deposits were identified through optical images by using three distinct light filters: plane-polarized light, cross-polarized light and full-wave retardation plate (λ plate). The use of the λ plate allowed us to distinguish the various dental tissues and cementum types (e.g. AC versus intrinsic cellular mixed stratified cementum) and to identify potential diagenetic alterations of the cementum (false increments, collagen leaching; cf. Stutz^124^).

## Supplementary information

Dataset 1

## Data Availability

All data generated during this study are presented herein and included in the Supplementary Information.
